# Supplementary material for: Identification of photoperiod-induced specific miRNAs in the adrenal glands of Sunite sheep (Ovis aries)
Source: Front Vet Sci. 2022 Jul 22;9:888207. doi: 10.3389/fvets.2022.888207 (PMC9354845; doi:10.3389/fvets.2022.888207)
Supplement: Supplementary file 1 [file Data_Sheet_1.ZIP › Supplementary file/miRDeep2 file.zip!/miRDeep2 file/Novel_Structure/LP42A2/Novel_647.pdf]

Provisional ID	:	NC_019473.2_27013
Score total	:	19.4
Score for star read(s)	:	3.9
Score for read counts	:	11.3
Score for mfe	:	2.6
Score for randfold	:	1.6
Score for cons. seed	:	
Total read count	:	34
Mature read count	:	33
Loop read count	:	0
Star read count	:	1

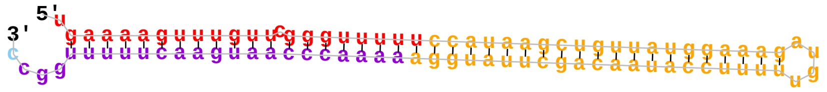

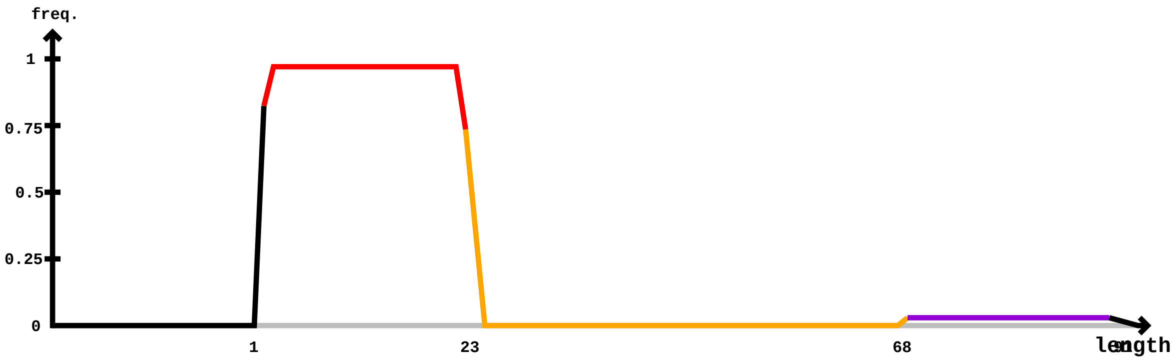


```
                         Mature                                                                        Star
5'- gcccacagacacuggguuggugaaaaguuuguucggguuuuuccauaagcuguuauggaaagauguuuuuccauaacagcuuauggaaaaacccaaugaacuuuuuggccaa   -3'    obs
    gcccacagacacuggguuggugaaaaguuuguucggguuuuuccauaagcuguuauggaaagauguuuuuccauaacagcuuauggaaaaacccaaugaacuuuuuggccaa          exp
    (((((.......)))))((((.(((((((((((.((((((((((((((((((((((((((((....)))))))))))))))))))))))))))))))))))))))..)))).   reads   mm   sample
    ....................ugaaaaguuuguucggguuuu.......................................................................   8       0    seq
    ....................ugaaaaguuuguucggguuuuu......................................................................   19      0    seq
    ....................ugaaaaguuuguucggguuuuuc.....................................................................   1       0    seq
    .....................gaaaaguuuguucggguuuuu......................................................................   5       0    seq
    .......................................................................................aaaacccaaugaacuuuuuggc...   1       0    seq
```
